# Ersodetug for refractory hypoglycemia due to malignant insulin-secreting tumors

**DOI:** 10.1210/clinem/dgag318

**Published:** 2026-08-06

**Authors:** Jonathan Strosberg, Sean M Brown, Shagufta Shaheen, Marilyn Tan, Jairo Arturo Noreña, Armen I Yerevanian, Linda Lester, Leah M Wilson, Ole-Petter R Hamnvik, Johannes Hofland, Jasmine K Sidhu, Gopal Saha, Davelyn E Hood, Brian K Roberts, Jonathan M Dreyfuss, Mary-Elizabeth Patti

**Affiliations:** Department of Gastrointestinal Oncology, Moffitt Cancer Center, Tampa, FL 33612, USA; Research Division, Joslin Diabetes Center (Harvard Medical School), Boston, MA 02115, USA; Division of Endocrinology, Stanford University Medical Center, Stanford, CA 94305, USA; Division of Endocrinology, Stanford University Medical Center, Stanford, CA 94305, USA; Division of Endocrinology, Stanford University Medical Center, Stanford, CA 94305, USA; Diabetes Unit, Massachusetts General Hospital (Harvard Medical School), Boston, MA 02114, USA; Division of Endocrinology, Diabetes and Clinical Nutrition, Oregon Health & Science University, Portland, OR 97239, USA; Division of Endocrinology, Diabetes and Clinical Nutrition, Oregon Health & Science University, Portland, OR 97239, USA; Division of Endocrinology, Diabetes, and Metabolism, Brigham and Women's Hospital (Harvard Medical School), Boston, MA 02115, USA; Department of Internal Medicine, Erasmus Medical Center, 3000 CA Rotterdam, Netherlands; Rezolute Inc., Redwood City, CA 94065, USA; Rezolute Inc., Redwood City, CA 94065, USA; Rezolute Inc., Redwood City, CA 94065, USA; Rezolute Inc., Redwood City, CA 94065, USA; Research Division, Joslin Diabetes Center (Harvard Medical School), Boston, MA 02115, USA; Research Division, Joslin Diabetes Center (Harvard Medical School), Boston, MA 02115, USA

**Keywords:** tumor hyperinsulinism, insulinoma, insulin receptor, monoclonal antibody, ersodetug, paraneoplastic hypoglycemia

## Abstract

**Background:**

Refractory hypoglycemia is common in malignant insulin-secreting tumors and is associated with substantial morbidity. Ersodetug is a fully human monoclonal antibody that allosterically attenuates insulin receptor signaling with the potential to treat various forms of hyperinsulinism (HI). We retrospectively report outcomes from compassionate use of ersodetug in 8 individuals with refractory hypoglycemia due to tumor HI.

**Methods:**

Ersodetug was administered intravenously at 6 or 9 mg/kg every 1-2 weeks initially, followed by a frequency of every 2-5 weeks, as appropriate. Glycemic, functional, and safety outcomes were analyzed.

**Results:**

Eight adults (4M/4F; 24-74 years; Eastern Cooperative Oncology Group [ECOG] 1-3) received ersodetug for insulin-secreting tumors (metastatic insulinoma, n = 7; cervical neuroendocrine carcinoma, n = 1). No drug-related serious adverse events were observed. Most patients experienced improved glycemic control, including discontinuation of parenteral glucose in 6 of the 7 applicable patients (median, 4.5 days) and a 35.6% relative reduction from baseline in time in hypoglycemia (<70 mg/dL) by continuous glucose monitoring (mean 12.4-8.0%; pseudo-median paired change of -3.8 percentage points [90% CI, −8.2 to −1.1]; *P* = .02). These glycemic improvements permitted hospital discharge and reductions in other antihypoglycemic therapies beyond glucose infusion rate (pseudo-median change, −2.5; 90% CI, −3.5 to −1.0; *P* = .02), with associated improvements in ECOG status. The median treatment duration with ersodetug (11.5 months, range 5-17) tended to correspond to patient lifespan in the setting of metastatic disease.

**Conclusion:**

Ersodetug therapy resulted in reduced hypoglycemia burden, ability to discontinue parenteral glucose infusion, allowing discharge from hospital, use of fewer antihypoglycemic therapies, and improved ECOG performance status in individuals with severe, refractory tumor HI.

Tumor hyperinsulinism (tumor HI) is caused by insulin or insulin-like growth factor-2 secretion by tumors, leading to life-threatening, refractory hypoglycemia (1-3). There are historically 2 main categories of rare neoplasms associated with tumor HI including pancreatic neuroendocrine tumors, which secrete excessive insulin, and solid tumors of mesenchymal or epithelial origin that overproduce incompletely processed insulin-like growth factor-2 variants (1, 2). Both pathophysiologic mechanisms lead to overactivation of the insulin receptor, resulting in insulin receptor–mediated hypoglycemia (1, 2).

While both pancreatic neuroendocrine tumors and insulin-like growth factor-2 producing solid tumors are rare, with estimated incidence rates of 4-15 cases per million individuals per year (4, 5), hypoglycemia is the universal functional consequence of tumor HI and is often the presenting feature (6). The clinical manifestations of hypoglycemia are marked by adrenergic, cholinergic, and neuroglycopenic symptoms such as tremors, diaphoresis, confusion, seizures, and loss of consciousness, and may lead to life-threatening consequences (3, 7). In individuals who are not candidates for or have not responded to interventions such as surgery or chemotherapy, effective glycemic control becomes the most urgent priority of care. Despite this clinical urgency, a significant unmet need remains, as current standard-of-care therapies to reduce hypoglycemia often fail to achieve sustained glycemic stability and are associated with high morbidity (hospitalization, poor functional status, etc.) (7, 8), underscoring the need for more effective treatment options.

The activity of insulin on target tissues is mediated by insulin receptor signaling, which causes increased glucose uptake into tissues, glycogen synthesis, suppression of gluconeogenesis, and suppression of free fatty acids and ketones (9, 10). Ersodetug, a fully human insulin receptor–modulating antibody, engages an allosteric site on the insulin receptor to selectively and reversibly reduce insulin binding and signaling in a dose-dependent manner, leading to reduced insulin signaling (11, 12). In healthy volunteers and individuals with congenital HI, ersodetug displays dose-proportional pharmacokinetics after intravenous administration, with peak serum concentrations observed within approximately 2 hours, an effective half-life of about 15 days (terminal elimination half-life 15-28 days), modest accumulation with repeat dosing, and concentrations that are predicted to approach or exceed 90% of steady-state within roughly 4-8 weeks depending on dosing frequency, with steady-state generally achieved by about 12 weeks. Because of its unique mechanism of action downstream from the pancreas and its sustained, predictable exposure profile with repeat dosing, ersodetug offers a novel approach to treating hypoglycemia caused by various congenital and acquired forms of HI (11-13). Its pharmacokinetic characteristics informed selection of a 90-day evaluable window during which drug concentrations were expected to be at steady-state for most individuals.

Following a previously published individual case report (14), this case series retrospectively reports on the experience of 8 individuals with refractory hypoglycemia due to tumor HI (metastatic insulin-producing tumors) who were treated with ersodetug on a compassionate use basis via an expanded access program (EAP).

## Materials and methods

### EAP overview

The EAP was initiated to provide compassionate access to ersodetug for individuals with severe, refractory hypoglycemia due to HI. Enrollment was initiated by a treating physician through a named patient request (single-patient investigational new drug application) to the country health authority and was approved by local institutional review board, if required.

Eight individuals with refractory hypoglycemia secondary to HI whose glycemic needs were not adequately managed by standard-of-care therapies were enrolled in the EAP beginning in November 2022; data were collected until the data cutoff of August 2025. Written informed consent was obtained from all individuals before treatment initiation. Individuals were eligible regardless of tumor type, prior therapies, or comorbidities, provided they were clinically stable for infusion. All listed diagnoses were confirmed by relevant laboratory, imaging and pathology over the treatment courses and prior to initiating ersodetug.

The first 2 individuals were enrolled under an earlier compassionate-use program framework; a subsequent, tumor HI–specific framework was implemented during the course of the third patient's treatment and applied to all remaining individuals. The updated framework incorporated a higher weekly dose that had shown superior effectiveness in early experience, and further details of the protocol-version transitions are provided in the supplemental materials (15).

For Patient 1, the continuous glucose monitoring (CGM) and glucose infusion rate (GIR) data for the 90-day pre- and post-treatment windows presented herein also were previously reported in a single case report (14); all other efficacy data for this patient are new.

### Treatment and procedures

Ersodetug was administered intravenously over 30 minutes at an initial dose of 6 or 9 mg/kg, given either weekly or every other week initially, followed by a frequency of every 2-5 weeks, as appropriate, at the discretion of the treating physician. Dose adjustments, including titration and changes in dosing frequency, were made based on glycemic response, tolerability, and clinical course. After exploratory dose-ranging in the first 2 participants, the initiation dose regimen was standardized in subsequent participants to 9 mg/kg weekly, followed by de-escalation as indicated. One participant required escalation from 9 to 12 mg/kg every other week. Table S2 details dosing regimens for each participant (15).

Supportive therapies, including use of ongoing standard-of-care antihypoglycemic treatments, were noted and recorded. Individuals resumed tumor-directed therapies or procedures as deemed appropriate by the multidisciplinary clinical care team, and these interventions were systematically recorded as part of the observational dataset.

### Data collection and assessments

In this retrospective analysis, observational data were collected until August 2025 (or earlier treatment discontinuation) in all individuals who received at least one infusion of ersodetug and had available follow-up data. For standardization of glycemic assessments, baseline was defined as the period up to 90 days prior to ersodetug initiation. The evaluable period was defined as the time from ersodetug initiation until the data cutoff (August 2025) or treatment discontinuation.

Assessments of efficacy focused on changes in GIR (since most participants initially required parenteral dextrose to correct refractory hypoglycemia) and also included assessments of hypoglycemia by CGM (described below), number of hospitalization days in the month before and after ersodetug initiation, and overall survival. Eastern Cooperative Oncology Group (ECOG) performance status was used to assess functional ability. The ersodetug dosing regimen, treatment duration, and safety observations (serious adverse events and drug-related serious adverse events) were also recorded and assessed. Disease progression was not reported as a treatment-emergent adverse event, as per EAP treatment guidelines.

### CGM data

CGM data were analyzed for mean sensor glucose and percent of time in predefined glycemic ranges (eg, <70 mg/dL, <54 mg/dL, and >180 mg/dL). Baseline for CGM purposes was defined as the period up to 90 days before the first dose of ersodetug, and the evaluable window was defined as the period up to 90 days after initiation; longer follow-up CGM data were included in a sensitivity analysis. Most individuals were receiving parenteral dextrose during baseline, which by intent likely ameliorated hypoglycemia, thus masking the severity of baseline glycemic measures by CGM. Therefore, changes from baseline in CGM should be interpreted accordingly. Details of data processing, range definitions, and statistical methods are provided in the Supplemental Appendix (15).

### Statistical analysis

Changes in percent time in glycemic ranges and medication counts were assessed from paired comparisons of the baseline period (up to 90 days) with the post-initiation evaluable period (up to 90 days) using the Wilcoxon-signed rank test in R. Results are reported using descriptive statistics, including the observed means at both baseline and the evaluable period, relative change from baseline (%), and the pseudo-median (termed the “median”) and 90% confidence interval (CI) for the individual absolute paired changes (95% intervals could not be constructed due to the sample size [N = 8] and discrete nature of the test). Strength of statistical evidence was evaluated using nominal *P*-values, but no formal or prespecified hypothesis testing was conducted due to the retrospective nature of the report and limited sample size. Sex was not considered as a factor in the statistical analysis due to the small sample size and resulting lack of statistical power to support stratified or adjusted analyses, and because it was not considered relevant to the mechanism of disease or action of ersodetug. A statistical analysis plan with additional details of the statistical methods was developed after primary data analyses and is available in the Supplemental Appendix (15).

### Data availability

Restrictions apply to the availability of some or all data generated or analyzed during this study to preserve patient confidentiality or because they were used under license. The corresponding author will on request detail the restrictions and any conditions under which access to some data may be provided.

## Results

### Participant disposition

A summary of demographics and clinical baseline characteristics is provided in Table 1. Eight individuals with equal gender distribution, aged 24-74 years, were enrolled in the EAP in the United States (n = 7) and the Netherlands (n = 1) and received ersodetug for refractory hypoglycemia due to tumor HI. All individuals were White; 7 identified as non-Hispanic and one as Hispanic. Seven individuals had metastatic insulinoma and one had neuroendocrine carcinoma of the cervix. The cohort was characterized by predominantly metastatic disease with substantial hepatic involvement and variable but often abnormal liver biochemistry, along with renal function ranging from normal to mildly impaired at the time of ersodetug initiation. Table S1 provides background information on insulinoma and the representativeness of this case series (15).

**Table 1:** Demographics and clinical baseline characteristics

	Patient 1*^a^*	Patient 2	Patient 3	Patient 4	Patient 5	Patient 6	Patient 7	Patient 8
**Demographics**								
Gender (M/F); age (years)	M; 55	F; 50	F; 50	F; 43	M; 74	M; 62	F; 74	M; 24
Country	U.S.	U.S.	U.S.	U.S.	U.S.	U.S.	U.S.	The Netherlands
Race/Ethnicity	White, Hispanic	White, Non-Hispanic	White, Non-Hispanic	White, Non-Hispanic	White, Non-Hispanic	White, Non-Hispanic	White, Non-Hispanic	White, Non-Hispanic
**Clinical baseline**								
Dx*^c^*	Metastatic Insulinoma	Metastatic Insulinoma	Neuroendocrine Carcinoma of the Cervix	Metastatic Insulinoma	Metastatic Insulinoma	Metastatic Insulinoma	Metastatic Insulinoma	Metastatic Insulinoma
Dx year	2021	2015	2022	2021	2023	2019	2022	2022
Tumor primary site	Pancreas	Pancreas	Cervix (neuroendocrine carcinoma)	Pancreas	Pancreas (primary pancreatic NET with liver metastases)	Pancreas (well-differentiated pancreatic NET)	Pancreas (well-differentiated NET in pancreatic body)	Pancreas (primary insulinoma diagnosed 2022; right-sided hemihepatectomy in 2024 at time of insulinoma)
Ki-67 (% and biopsy site)	8% (liver metastasis biopsy)	17.6% (liver resection specimen); primary pancreatic tumor Ki-67 9%	75% (liver metastasis biopsy)	5% (pancreatic primary tumor)	35% (liver metastasis; secondary tumor)	Unknown (fine-needle aspiration showing well-differentiated pancreatic NET)	42% (liver core needle biopsy; WHO grade 3 well-differentiated NET)	16% (pancreatic primary); 17.8% (liver metastasis)
Metastatic sites and burden	Multiple abdominal and multilobar hepatic lesions, abdominal pain	Liver, gastrohepatic lymph nodes, osseous metastases	Extensive metastatic burden (Multilobar liver and pelvic metastases)	Extesive metastatic burden (Liver, lymph nodes, bone)	Extensive liver tumor burden	Liver, periportal lymph nodes, para-aortic lymph nodes, low overall disease burden	Extensive bilobar hepatic metastatic burden; left hilar metastasis	Extensive liver tumor burden, involving an estimated 33% of the remaining left hemi-liver
Hepatic function at erosdetug initiation	Mild ↑ transaminases, low albumin	Marked ↑ transaminases, low albumin	Mild ↑ in alk phos, low albumin	Marked ↑ alk phos, low albumin	Mild ↑ alk phos, low albumin	Modest ↑ transaminases	Moderate ↑ alk phos,	Mild ↑ transaminases, and alk phos
ALT/AST (U/L)	81, 38	148, 144,	22. 17	20, 53	36, 26	87, 75	44, 20	40, 44
Alk phos (U/L)	105	620	173	677	108	104	255	161
Albumin (g/dL)	3.4	3.1	3.4	3.1	3.1	3.9	3.8	3.8
PT (sec)	NA	16.9*^b^*	18.4	12	11.3	11.5	13.9	NA
INR (ratio)	1.1	NA	NA	NA	1.0	1.0	1.2	1.1
Renal function at ersodetug initiation	Normal	Normal	Impaired	Mildly impaired	Normal	Normal	Normal	Mildly impaired
creatinine (mg/dL)	1.2	0.8	2.13	1.26	1.1	0.9	0.63	1.3
eGFR (mL/min/1.73 m^2^)	NA	90	49	54	>60	>60	93	73
Length of hospitalization prior to ersodetug (days)	28	NA (ambulatory)	15	49	34	NA (ambulatory)	4	16
Hospice planning Before ersodetug	Y	N	Y	N	N	N	N	N
Baseline ECOG	3	2	3	3	3	1	1	1
**Standard-of-care therapy history**								
No. of therapies used to manage hypoglycemia (medications and IV glucose) before ersodetug	4	6	4	5	4	2	3	2
Diazoxide	+	+	+	+	Previously Tried	Previously Tried	Previously Tried	+
SSA- short-acting (Octreotide/Pasireotide)	Previously Tried	+	+	Previously Tried	+	NA	Previously Tried	Previously Tried
SSA- long-acting (Lanreotide)	−	+	NA	+	NA	+	Previously Tried	NA
mTOR inhibitor (Everolimus)	+	+	NA	+	+	+	+	NA
Corticosteroid (Dexamethasone, Prednisone)	+	+	+	+	+	NA	+	NA
**IV glucose**								
GIR (mg/kg/min) at ersodetug initiation	6.0	7.0	5.1	6.2	4.9	NA	5.6	3.1

Abbreviations: ALP, alkaline phosphatase; ALT, alanine aminotransferase; AST, aspartate aminotransferase; Dx, diagnosis; ECOG, Eastern Cooperative Oncology Group performance status; eGFR, estimated glomerular filtration rate; GIR, glucose infusion rate; INR, International Normalized Ratio; mTOR, mammalian target of rapamycin; NA, not available/assessable; NET, neuroendocrine tumor; PT, prothrombin time; SSA, somatostatin analogue; WHO, World Health Organization.

^*a*^This patient was the first to receive ersodetug for tumor HI, with detailed results previously reported in a case report (14).

^*b*^13 days after infusion

^*c*^All listed diagnoses were confirmed by relevant laboratory, imaging and pathology over the treatment courses and prior to initiating ersodetug.

All individuals had received and were refractory to at least 3 standard-of-care therapies, including diazoxide, short- and long-acting somatostatin analogs, everolimus, and/or corticosteroids. Six of the 8 individuals were hospitalized at EAP entry (median, 28 days; range, 4-66 days), largely due to glycemic instability and the need for inpatient or intensive parenteral glucose support. Two individuals initiated ersodetug prior to hospitalization, including one receiving continuous carbohydrate supplementation via home TPN. Of the 7 individuals who were receiving parenteral glucose, including one on home TPN, baseline GIRs at ersodetug initiation ranged from 3.1 to 7.0 mg/kg/min. Four of the 8 individuals had a baseline ECOG score of 3, 1 had an ECOG of 2, and 3 had an ECOG of 1. Two individuals were under hospice consideration at baseline due to refractory hypoglycemia, progressive disease, and lack of remaining tumor-directed treatment options. The median treatment duration with ersodetug (11.5 months, range 5-17) tended to correspond to patient lifespan in the setting of metastatic disease.

Individual-patient case summaries are summarized in Table S3 (15).

### Glycemic efficacy outcomes

Six of 7 individuals on parenteral glucose support at baseline discontinued it within the 90-day post-therapy window (median time, 4.5 days), which allowed for subsequent reductions in other standard-of-care antihypoglycemic therapies. One individual on home TPN initiated ersodetug contemporaneously with ablation of primary hepatic metastases, ultimately achieving a 50% reduction in intravenous carbohydrate support after dose escalation of ersodetug to 9 mg/kg biweekly. The total number of therapies decreased from a mean of 3.8 to 1.6 during the 90 days after ersodetug initiation (median change, −2.5; 90% CI, −3.5 to −1.0; *P* = .02) (Fig. 1A and Table 2).

**Figure 1:**
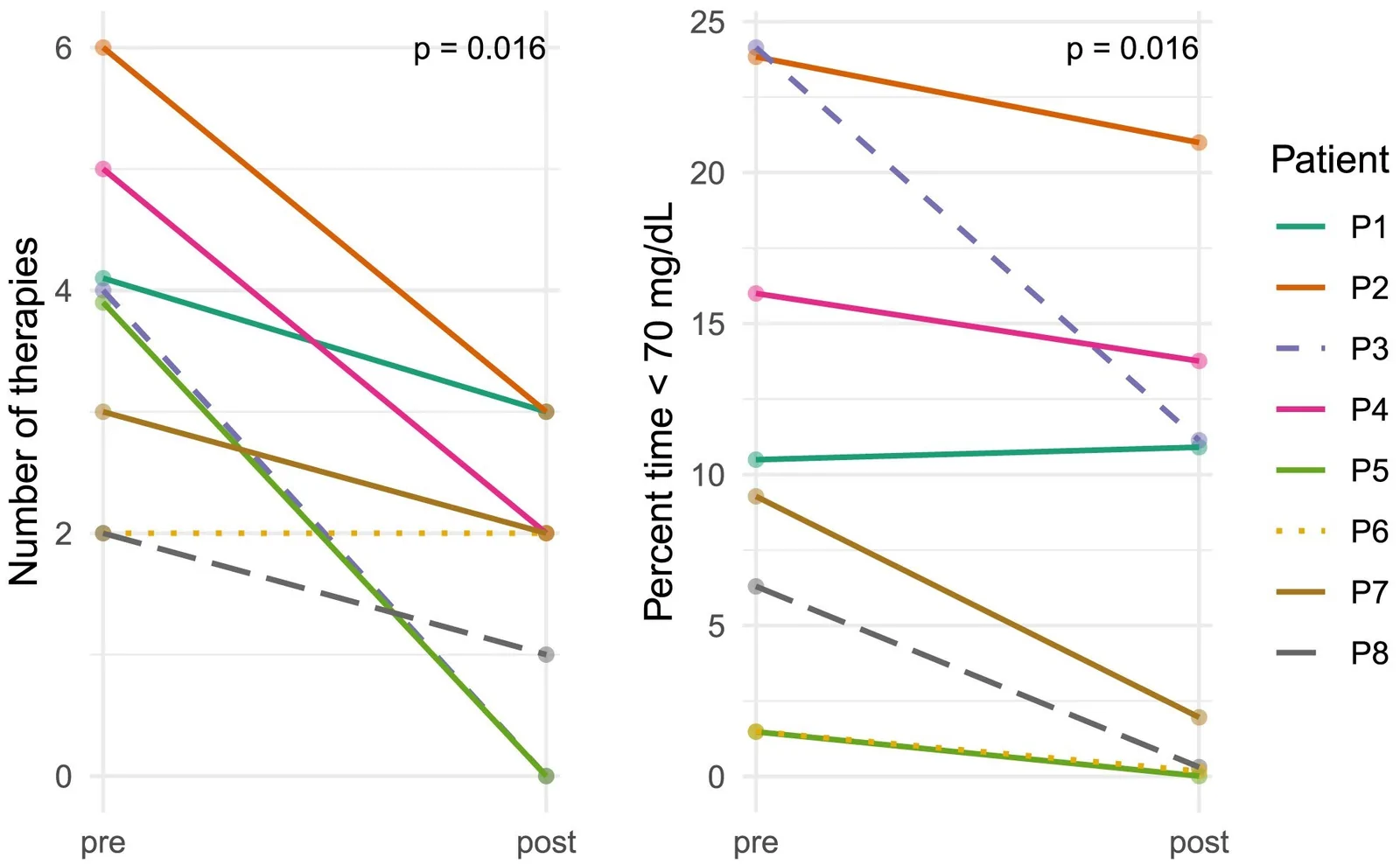
A. Number of therapeutic interventions used per patient, from 90 days before therapy to 90 days after initiation of therapy with ersodetug. B. Percent time with sensor glucose under 70 mg/dL, assessed over 24 hours, from 90 days before therapy to 90 days after initiation of therapy with ersodetug. For both, *P*-value for Wilcoxon-signed rank test of paired differences is provided. Lines are slightly offset to show overlapping patient data.

**Table 2:** Summary of clinical outcomes

	Patient 1	Patient 2	Patient 3	Patient 4	Patient 5	Patient 6	Patient 7	Patient 8
**Glycemic SOC management outcomes**	**Time to discontinuation after ersodetug initiation (days)**							
IV glucose	74 (slow taper)	139 (50% reduction)	4	5	2	NA	3	42
Diazoxide	111	10	4	21	NA	NA	NA	Ongoing
SSA—short acting (Octreotide/Pasireotide)	NA	18	4	NA	8	NA	NA	NA
SSA—long-acting (Lanreotide)	NA	Ongoing	NA	30	NA	Ongoing	NA	NA
mTOR inhibitor (Everolimus)	∼6 months	18	NA	Ongoing	8	Ongoing	Ongoing	NA
Corticosteroid (Dexamethasone, Prednisone)	∼7 months	∼4 months	4 days	Ongoing	NA	NA	Ongoing	NA
**Hospital discharge following ersodetug administration**								
Time to discharge from hospital (days)	84	NA	7	15	9	NA	9	1
Hospitalization days in month after ersodetug initiation	30	0	7	8	8	0	16	1
**Tumor-directed therapies within 8 weeks after initiating ersodetug**								
Chemotherapy: Yes/No; Therapy Name	Y; LuTate	Y; FOLFOX	Y; Pembrolizumab	Y; CAPTEM	Y; LuTate	NA	Y; FOLFOX	Y; CAPTEM
Surgery: Yes/No; Surgical procedure	N	Y; Whipple Surgery and Left Hepatectomy	Y; Total hysterectomy and bilateral oophorectomy	Y; Partial Pancreatectomy	N	N	N	Y; Hemi-hepatectomy
Other medical procedure: Yes/No; Procedure type	N	Y; HACE	N	Y; HAE	Y; HAE	Y; HAE	Y; HAE	NA
**Estimated ECOG status**								
3-month ECOG	0	2	0	0	0	0	1	0
**Survival**								
Overall survival (months)	14 months	5 months	5 months	14 months	17 months (living)	14 months (living)	6 months	10 months
Cause of death	Tumor progression	Tumor progression	Tumor progression	Tumor progression	NA	NA	MDS Progression/Sepsis	Tumor Progression

Abbreviations: CAPTEM, capecitabine + temozolomide; ECOG, Eastern Cooperative Oncology Group performance status; FOLFOX, 5-fluorouracil + oxaliplatin; HACE, hepatic arterial chemo-embolization; HAE, hepatic arterial embolization; LuTate, 177Lu-DOTATATE; MDS, myelodysplastic syndrome; mTOR, mammalian target of rapamycin; NA, not available/assessable; OS, overall survival; SOC, standard of care; SSA, somatostatin analog.

All 8 individuals had CGM data available. Individual-patient CGM data showing dosing, time in hypoglycemia (<70 mg/dL, panel B; <54 mg/dL, panel C), and concomitant antihypoglycemic therapies (including GIR) are depicted in Fig. S1–S8 (15). Aggregate CGM data are presented in Table 3.

**Table 3:** Summary of mean observed values and median changes from baseline in CGM parameters

	Baseline (90 days before initiation of ersodetug)	Evaluable period (90 days after initiation of ersodetug)	Relative change from baseline (%)	Median change from baseline	*P* value
**24 hours**					
Mean sensor glucose (mg/dL)	**117.0** (86.9, 157.6)	**138.0** (94.3, 189.5)	18.0	**21.6** (6.5, 36.1)	*0*.*02*
Time above 180 (%)	**10.1** (0.6, 29.3)	**22.7** (0.2, 56.1)	124.4	**13.5** (2.9, 24.3)	*0*.*02*
Time between 70 and 180 (%)	**77.4** (61.0, 95.0)	**69.2** (43.9, 87.3)	−10.6	**−8.2** (−20.2, 4.8)	0.08
Time below 70 (%)	**12.4** (1.7, 25.9)	**8.0** (0.0, 22.4)	−35.6	**−3.8** (−8.2, −1.1)	*0*.*02*
Time below 54 (%)	**2.6** (0.0, 5.2)	**1.9** (0.0, 6.8)	−29.2	**−0.5** (−2.9, 1.3)	0.38
**Day only (06:00 to 00:00)**					
Mean sensor glucose (mg/dL)	**117.2** (86.9, 155.7)	**139.5** (99.0, 191.2)	19.0	**21.8** (9.5, 36.9)	*0*.*008*
Time above 180 (%)	**10.4** (0.7, 31.7)	**23.2** (0.3, 57.7)	123.0	**12.9** (3.8, 23.7)	*0*.*02*
Time between 70 and 180 (%)	**76.8** (58.2, 95.0)	**69.4** (42.3, 92.6)	−9.6	**−6.2** (−19.7, 7.4)	0.25
Time below 70 (%)	**12.8** (0.8, 27.5)	**7.3** (0.0, 22.3)	−42.6	**−4.6** (−11.6, −0.7)	*0*.*02*
Time below 54 (%)	**2.7** (0.0, 4.5)	**2.0** (0.0, 7.2)	−26.8	**−0.7** (−2.8, 1.4)	0.46
**Night only (00:00 to 06:00)**					
Mean sensor glucose (mg/dL)	**116.4** (87.0, 163.3)	**134.2** (80.3, 196.6)	15.2	**15.6** (−3.1, 43.2)	0.11
Time above 180 (%)	**9.3** (0.0, 26.4)	**21.6** (0.0, 57.4)	132.1	**9.3** (0.6, 26.7)	*0*.*02*
Time between 70 and 180 (%)	**79.3** (69.4, 95.0)	**68.3** (42.0, 90.8)	−13.9	**−7.5** (−23.9, −1.0)	*0*.*02*
Time below 70 (%)	**11.3** (0.8, 26.0)	**10.0** (0.0, 28.8)	−11.8	**−1.1** (−4.5, 2.6)	0.25
Time below 54 (%)	**2.5** (0.0, 7.6)	**1.6** (0.0, 5.4)	−37.1	**−1.1** (−4.8, 2.4)	0.55

Pre- and post-therapy values are presented as mean (minimum, maximum). Median change values are presented as pseudo-median paired change (lower 90% confidence limit, upper 90% confidence limit). *P*-values were derived from two-sided Wilcoxon-signed rank tests (N = 8 pairs). Mean and median change values are presented in bold font. *P* < .05 are indicated by italic font.

Abbreviation: CGM, continuous glucose monitoring.

In the setting of reductions in GIR, mean sensor glucose increased from 117.0 to 138.0 mg/dL (median absolute change = 21.6 mg/dL; 90% CI, 6.5 to 36.1; *P* = .02). During the baseline period, the group had substantial time below 70 mg/dL (range, 1.7% to 25.9%; Fig. 1B), despite receipt of parenteral glucose for a prolonged period in the majority of cases. Seven of 8 individuals achieved a reduction in percent time in hypoglycemia, with a mean decrease from 12.4% at baseline to 8.0% after receiving ersodetug (35.6% relative reduction), equating to a median absolute change of −3.8% (90% CI, −8.2 to −1.1; *P* = .02) (Fig. 1B). Percent time in Level 2 hypoglycemia (<54 mg/dL) also trended numerically lower, with a mean decrease from 2.6% at baseline to 1.9% after receiving ersodetug (median absolute change of −0.5%; 90% CI, −2.9 to 1.3; *P* = .38). Similar patterns were observed for daytime-only sensor glucose values, with a median absolute decrease from baseline of −4.6% (90% CI, −11.6 to −0.7; *P* = .02). Reductions in overnight percent time in overall and Level 2 hypoglycemia were also observed, but statistical evidence was limited. Reflecting the shift toward higher sensor glucose values, ersodetug use also was associated with a numeric reduction in time in the target range (70-180 mg/dL) from a mean baseline of 77.4% to 69.2%, equating to a median absolute change of −8.2% (90% CI, −20.2 to 4.8; *P* = .08), and an increase in the percent time >180 mg/dL, from a mean of 10.1% at baseline to 22.7% (median absolute change = 13.5%; 90% CI, 2.9 to 24.3; *P* = .02). No hyperglycemia-related adverse events or need for insulin were reported.

In a sensitivity analysis that incorporated all available CGM data beyond the initial 90-day windows for patients with evaluable recordings, clinical patterns remained consistent with the primary analysis (see the Supplemental Appendix for more details) (15).

### Additional clinical outcomes

The median time to hospital discharge after initiation of ersodetug was 9 days (range, 1-84); all patients hospitalized at baseline (n = 6) were discharged to home for treatment, including 2 who were previously considering hospice-level care. Within 8 weeks of ersodetug initiation, nearly all individuals (n = 7) were able to proceed with tumor-directed therapies, including chemotherapy (n = 7), surgery (n = 4), and other medical procedures such as hepatic arterial embolization (n = 5).

ECOG performance status was evaluated 3 months after initial ersodetug administration; 6 individuals had a score of 0, 4 of whom improved from a baseline score of 3.

Most individuals (n = 6/8) were able to decrease the dosing frequency of ersodetug after hospital discharge. The median treatment duration with ersodetug of 11.5 months (range 5-17 months; ongoing in 2 individuals) also corresponded to patient lifespan and overall survival in the setting of metastatic disease. Of the individuals who died (n = 6), tumor progression and sepsis were cited as the cause, unrelated to the investigational drug.

### Safety outcomes

Ersodetug was generally safe and well tolerated among all individuals and across all dose levels and regimens. There were no drug-related deaths, adverse drug reactions, discontinuations due to adverse events, treatment-emergent clinically significant laboratory abnormalities indicative of organ toxicity, or other dose-limiting toxicities, as determined by treating physicians and reviewed by the sponsor's safety committee. As depicted in Table 4, 3 of 8 individuals experienced serious adverse events, all attributed to underlying tumor progression or tumor-directed therapies. A more comprehensive summary of serious treatment-emergent adverse events by system organ class and preferred term is provided in Table S4 (15).

**Table 4:** Overview of treatment-emergent serious adverse events

	Pooled cohort (n = 8) number (%)
Individuals with serious TEAEs (SAEs), n (%)	3 (37.5%)
Total SAEs	4
Individuals with PI-judged study drug-related SAEs, n (%)	0 (0%)
Individuals with SAE by severity, n (%)	
Grade 1/Mild	1 (12.5%)
Grade 2/Moderate	0 (0%)
Grade 3/Severe	2 (25%)
Grade 4/Life-threatening	1 (12.5%)
Grade 5/Death	0 (0%)

Abbreviations: PI, principal investigator; SAE, serious adverse event; TEAE, treatment-emergent adverse event.

## Discussion

In this case series of individuals with refractory hypoglycemia due to tumor HI, ersodetug was safely administered on a compassionate use basis and was associated with clinically meaningful and significant improvements in glycemic control, including discontinuation of parenteral glucose in most individuals as well as a 35.6% relative reduction in sensor values below 70 mg/dL by CGM, in the setting of reductions in other medical therapies for hypoglycemia. A corresponding increase in time above 180 mg/dL was observed, but without associated clinical impact.

With improvements in glycemic control, all individuals experienced associated functional benefits, including hospital discharge in previously prolonged inpatient cases, and marked improvements in ECOG performance status. These functional gains enabled most individuals to pursue tumor-directed therapies, including chemotherapy, surgery, and hepatic arterial embolization, underscoring the importance of glycemic stabilization as a prerequisite for oncologic care in this population.

In this expanded-access cohort, ersodetug was evaluated in individuals with severe, refractory hypoglycemia due to tumor-related HI, typically representing an end-of-life phase of the disease with limited therapeutic options and poor short-term prognosis. Contemporary series indicate that patients with metastatic insulinoma or other forms of tumor HI often have substantially longer 5-year survival (16-18), underscoring that hypoglycemia is the defining clinical burden across the broader disease spectrum, from earlier, milder presentations to late-stage disease (6). Because hypoglycemia in this setting is fundamentally driven by inappropriate insulin-mediated glucose disposal, the reductions in glucose infusion requirements and improvements in CGM-based hypoglycemia metrics observed here would be expected to translate into meaningful outpatient glycemic benefit in patients with less advanced disease, supporting the potential applicability of ersodetug beyond this severely ill cohort.

Glycemic stabilization was achieved across dosing regimens, with a more pronounced response observed in individuals initiated at 9 mg/kg weekly. More protracted responses to ersodetug and longer times to hospital discharge in initially treated individuals were associated with lower starting doses and less frequent administration of ersodetug (6 mg/kg and/or biweekly administration).

Current management of tumor HI underscores the limited efficacy and tolerability of available options and provides important context for the present findings. While surgical resection can be curative in localized disease, many patients require chronic medical therapy due to unresectable, metastatic, or multifocal tumors. Medical approaches are primarily directed at controlling hypoglycemia rather than the underlying tumor and include diazoxide, the only approved therapy for HI, and somatostatin analogs, both of which are widely used but often provide incomplete or transient benefit (19-23). Somatostatin analogs, in particular, are limited by tachyphylaxis and may paradoxically worsen hypoglycemia due to drug-induced reductions in glucagon secretion (1, 20, 24). Everolimus, although approved as a tumor-directed therapy for neuroendocrine tumors, is sometimes used for glycemic control but carries a substantial risk of adverse events, including immunosuppression and pneumonitis (25-27). Supportive strategies such as frequent carbohydrate intake or continuous parenteral dextrose infusion are often required but are burdensome and do not provide durable control, and parenteral infusions may prevent hospital discharge. In this context, the favorable glycemic and functional outcomes observed with ersodetug in the present series—particularly the reduction in hypoglycemia, decreased reliance on parenteral glucose, and facilitation of tumor-directed therapy—suggest that targeted approaches to directly counteract insulin receptor–mediated hypoglycemia may address a critical gap in the current treatment paradigm and offer a more effective option for patients requiring sustained glycemic control.

We acknowledge both strengths and limitations of our study. Strengths of the study include the long duration of clinical follow-up (median 11.5 months) and the analysis of clinically important outcomes including duration of hospitalization, glucose infusion rate, ECOG status, and safety outcomes. Moreover, analysis of sensor glucose using CGM allowed more comprehensive analysis of glycemic responses to ersodetug. Limitations include small sample size, retrospective reporting of observational data from an EAP, lack of a separate control group, nonstandardized management, concomitant treatments, incomplete data capture, and known limitations of CGM accuracy in hypoglycemia. Given the substantial interindividual variation in clinical stage, tumor burden, and magnitude of hyperinsulinemia and hypoglycemia among patients with tumor-associated hyperinsulinemia, the efficacy of ersodetug may vary in a larger cohort of patients, and individualized assessment of responses will be important.

In summary, this report provides important evidence supporting the use of ersodetug to achieve rapid glycemic stabilization and improved functional status in refractory tumor HI and provides important clinical context ahead of the ongoing phase 3 clinical trial (NCT06881992) for this form of HI.

## Resource availability

### Lead contact

Requests for reprints, further information, and resources should be directed to and will be fulfilled by the lead contact, Dr. Brian K. Roberts, who can be contacted at brian@rezolutebio.com.

### Materials availability

This clinical program did not produce any unique materials.

## Disclosures

B.K.R., D.E.H., J.K.S., and G.S. are employed by and shareholders in Rezolute, Inc. L.M.W. has received research support from Rezolute, Inc. and Dexcom; and acknowledges support from DK129382, DK122583 and DK120367, unrelated to the present report. M.E.P. acknowledges support from DK036836 (Joslin Diabetes Center DRC), and from DK137518 and DK106193, unrelated to the present report. Her institution receives funds for clinical trial investigation from Recordati and Amylyx, unrelated to the present report.

## Data Availability

Restrictions apply to the availability of some or all data generated or analyzed during this study to preserve patient confidentiality or because they were used under license. The corresponding author will on request detail the restrictions and any conditions under which access to some data may be provided.
