# Correction to: “Ersodetug for Refractory Hypoglycemia Due to Malignant Insulin-Secreting Tumors”

**DOI:** 10.1210/clinem/dgag360

**Published:** 2026-09-01

In the above-named article by Strosberg J, Brown SM, Shaheen S, Tan M, Noreña JA, Yerevanian AI, Lester L, Wilson LM, Hamnvik OR, Hofland J, Sidhu JK, Saha G, Hood DE, Roberts BK, Dreyfuss JM, Patti M-E (*J Clin Endo Metab*. 2026; doi: 10.1210/clinem/dgag318), there was an error in the reference section.

In the originally published article, the link to the supplementary material in reference 15 was inadvertently deleted. In the corrected article, a link to the supplementary material in reference 15 has been provided at DOI: 10.5281/zenodo.20147071.

The article has been corrected online.

The publisher regrets the error.

doi: 10.1210/clinem/dgag318

